# Multiple Regionalized Genes and Their Putative Networks in the Interpeduncular Nucleus Suggest Complex Mechanisms of Neuron Development and Axon Guidance

**DOI:** 10.3389/fnana.2021.643320

**Published:** 2021-02-16

**Authors:** Isabel M. García-Guillén, Antonia Alonso, Luis Puelles, Faustino Marín, Pilar Aroca

**Affiliations:** Department of Human Anatomy and Psychobiology, Faculty of Medicine, Regional Campus of International Excellence “Campus Mare Nostrum”, University of Murcia and IMIB-Arrixaca, Murcia, Spain

**Keywords:** interpeduncular nucleus, axon guidance, neuronal migration, ephrin, cadherin, interactome, rostral hindbrain, transcription factors

## Abstract

The interpeduncular nucleus (IPN) is a highly conserved limbic structure in the vertebrate brain, located in the isthmus and rhombomere 1. It is formed by various populations that migrate from different sites to the distinct domains within the IPN: the prodromal, rostral interpeduncular, and caudal interpeduncular nuclei. The aim here was to identify genes that are differentially expressed across these domains, characterizing their putative functional roles and interactions. To this end, we screened the 2,038 genes in the Allen Developing Mouse Brain Atlas database expressed at E18.5 and we identified 135 genes expressed within the IPN. The functional analysis of these genes highlighted an overrepresentation of gene families related to neuron development, cell morphogenesis and axon guidance. The interactome analysis within each IPN domain yielded specific networks that mainly involve members of the ephrin/Eph and Cadherin families, transcription factors and molecules related to synaptic neurotransmission. These results bring to light specific mechanisms that might participate in the formation, molecular regionalization, axon guidance and connectivity of the different IPN domains. This genoarchitectonic model of the IPN enables data on gene expression and interactions to be integrated and interpreted, providing a basis for the further study of the connectivity and function of this poorly understood nuclear complex under both normal and pathological conditions.

## Introduction

The interpeduncular nucleus (IPN) is a highly conserved limbic structure in the vertebrate brain, a compact composite of three rostrocaudal domains and diverse sub-nuclei. It has a sub-pial location across the ventral midline of the rostral (prepontine) hindbrain and it is generated by diverse neuronal populations that migrate there from different dorsoventral sites (Lorente-Cánovas et al., 2012). The main input to the IPN comes from the medial habenula (MHb) via the fasciculus retroflexus. In turn, the IPN establishes widespread projections that either ascend to limbic structures or descend to other brainstem nuclei (Groenewegen et al., 1986; Quina et al., 2017). Functionally, the MHb-IPN system is involved in several behavior-related activities, such as learning and memory, sleep, motor activity, stress, affective states (Klemm, 2004; Hikosaka, 2010) and mood-related psychiatric conditions (McLaughlin et al., 2017).

The IPN has a complex tridimensional organization, both cyto- and chemoarchitectonically, as might be expected by its multiple connections to the rest of the brain (Quina et al., 2017). According to the IPN model proposed in the chick embryo (Lorente-Cánovas et al., 2012), this nuclear complex is comprised of three main, rostrocaudally abutting domains that are located in contiguous segmental (neuromeric) units: a domain in the isthmus (Ist), the *prodromal nucleus* (Pro); a domain in rostral rhombomere 1 (r1-r), the *rostral IPN nucleus* (IPR); and a domain in the caudal rhombomere 1 (r1-c), the *caudal IPN nucleus* (IPC). Both the IPR and IPC are further divided along the mediolateral and dorsoventral axes into several sub-nuclei (Lorente-Cánovas et al., 2012). Subsequently, it was shown that this a nucleus follows a similar developmental course in mice (Moreno-Bravo et al., 2014; Ruiz-Reig et al., 2019; García-Guillén et al., 2020).

Several neuronal populations have been identified in these IPN structures, characterized by the expression of the transcription factors (TFs) *Nkx6.1*, *Pax7*, *Otp*, or *Otx2* (Lorente-Cánovas et al., 2012). These cell populations express these TFs throughout the development of the IPN, allowing their origin, migratory pathways and final fates in the nucleus to be determined.

Recently, the *Irx2* TF was identified as a novel marker of an IPN population (García-Guillén et al., 2020). Moreover, the migration of the *Irx2*^+^, *Nkx6.1*^+^, *Pax7*^+^, *Otp*^+^, and *Otx2*^+^ IPN populations was shown to be differentially regulated by the Netrin-1/DCC signaling system. Indeed, the migration of all these IPN neurons, which normally express the Netrin-1 receptor DCC, is severely disrupted in a *Dcc* knock-out mouse model (García-Guillén et al., 2020).

Due to its structural and histogenetic complexity, it would be expected that many other TFs would be expressed in the IPN, as well as other genes potentially involved in neural development. Therefore, we carried out a high-throughput search for genes that are expressed in the developing IPN, focusing on a late gestational stage when the migratory processes involved in its constitution have virtually been completed and the gross morphology of the IPN is evident. We performed this data mining on the Allen Developing Mouse Brain (ADMBA) database^1^. Through this analysis we identified 135 genes expressed in the developing mouse IPN, characterizing their expression relative to the IPN model. We also carried out a mining analysis that helped identify overrepresented functions in this structure, such as axon guidance and cellular morphogenesis, as well as a variety of putative interactions for each compartment within the IPN.

## Materials and Methods

### Mining of the Allen Brain Database

The ADMBA database holds high resolution *in situ* hybridization (ISH) imaging data across four prenatal and three early postnatal time points for 2,107 genes that are functionally relevant to brain development and/or disorders (Thompson et al., 2014).

We retrieved the image series of E18.5 embryos that processes data for 2,038 genes, with 69 genes discarded due to the lack of ISH data at this stage or damage to the IPN tissue. We screened these images visually, selecting the genes with significant expression within the IPN. This analysis was initially carried out by two of the authors independently, whose preliminary results were discussed to reach a consensus. Our criteria to select genes was that they displayed discrete expression patterns, with positive and negative regions visible within the IPN. As a result, we identified 135 genes (Supplementary Table 2) that were differentially expressed in the IPN, downloading their image series for further analysis. We then also analyzed the expression of the 135 genes selected in P4 brains and compared this to that observed at E18.5 (Supplementary Table 3).

**FIGURE 1:**
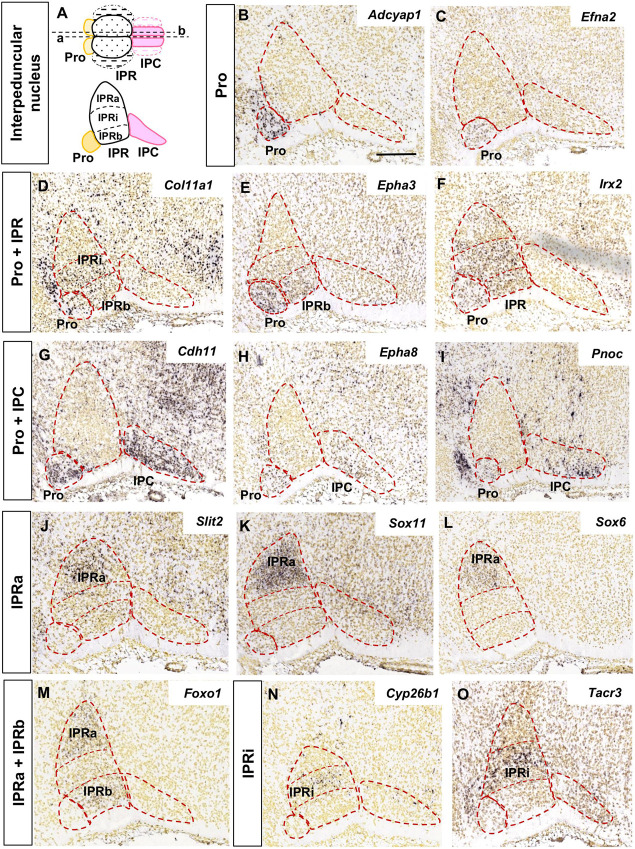
Regional gene expression in the IPN at E18.5 (I). **(A)** Schemes representing the dorsal and lateral views of the IPN with its main sub-divisions. In the dorsal view, a, b represent the median and paramedian planes of the sections analyzed, respectively. The dashed areas correspond to the lateral sub-divisions of the IPN that were not included in this study. **(B–O)** and Figure 2 show representative parasagittal sections centered on the IPN in the rostral hindbrain, highlighting the regionalized expression of genes. The rostral end is situated to the left in all images. The different expression patterns, corresponding to combinations of the IPN sub-divisions, are defined in the vertical boxes and followed by images of representative genes fitting each pattern. The abbreviations used are specified in the main text. Scale bars = 200 μm.

**FIGURE 2:**
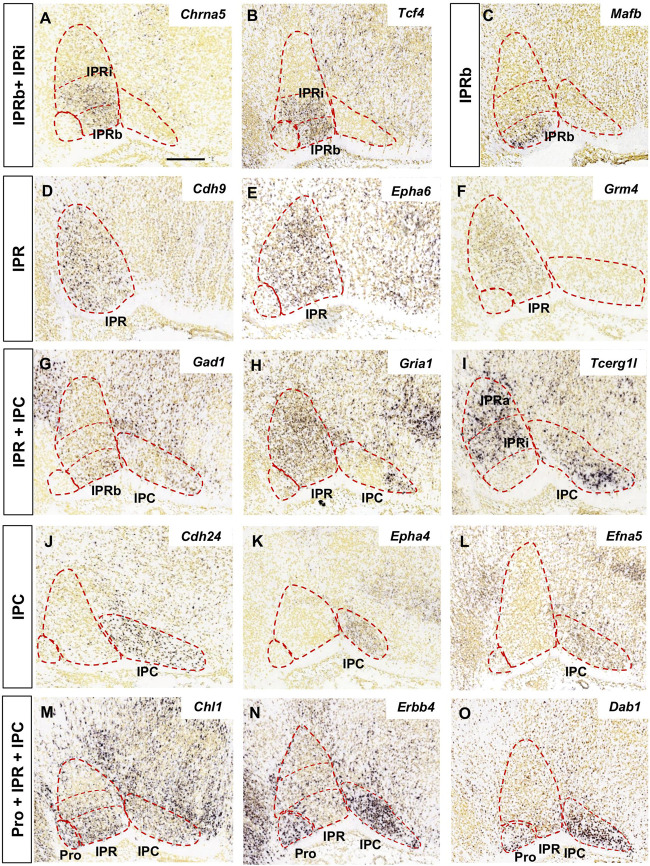
Regional gene expression in the IPN at E18.5 (II). Additional expression patterns (vertical boxes) followed by images of their corresponding genes **(A–O)** as displayed in Figure 1. Scale bars = 200 μm.

The analysis of the images focused on the median and paramedian sagittal sections from each brain where the IPN can be detected, using two to four sections depending on the experiment. In Figures 1, 2 the images were cropped, rotated and centered on the IPN, in accordance with the sagittal scheme shown in Figure 1A.

### Gene Ontology (GO) Classification and Statistical Overrepresentation Test

The genes selected were classified into Gene Ontology (GO) families using the BioMart tool, accessed through the Ensembl web^2^. This ontology^3^ uses a set of terms to describe biological functions and it associates genes or gene products with a GO term (Ashburner et al., 2000).

A statistical overrepresentation test was performed on the PANTHER Classification System^4^ using PANTHER version 15.0 released on February 14, 2020 (Mi et al., 2019a), along with the PANTHER GO-slim Biological Process and Molecular Function and Cellular Component datasets (Mi et al., 2019b). This test was applied to each functional category to determine whether there was a significant overrepresentation of any gene in the test list (of 135 selected genes) relative to the reference list (the initial 2,038 genes). The analysis was performed using the Fisher’s Exact test, applying a False Discovery Rate (FDR) correction. We limited our results to those functions displaying the strongest significance (*P* < 0.00005).

### Generation of Protein-Protein Interaction (PPI) Networks

We analyzed the functional protein association networks with the STRING database^5^, using STRING version 11.0b released on October 17, 2020 (Szklarczyk et al., 2019). This tool uses a combination of prediction approaches, integrating several sources of information (text mining, experiments, databases, co-expression, neighborhood, gene fusion, and co-occurrence). We retrieved the networks at a high level of confidence (0.700) based on all the prediction methods mentioned.

## Results

### Genes With Regional Expression in the IPN

To date, the *Nkx6.1, Pax7, Otx2, Irx2*, and *Otp* genes have been used to analyze IPN regionalization, which enable the migratory processes, and the principal Pro, IPR and IPC sub-divisions to be visualized at successive stages (Supplementary Figures 1, 2). Here we have looked for additional markers of IPN populations by searching the ADMBA database, which contains data from ISH experiments on generally well-oriented sagittal sections that allow the IPN nuclei to be identified. This screening yielded 135 genes that were differentially expressed across these nuclei at E18.5. As a first approach to characterize these genes, we distributed them into GO families that might be relevant to our working model in terms of different aspects of neural development (Supplementary Table 1): *regulation of transcription, DNA-templated* (GO: 0006355), *neuron projection guidance* (GO: 0097485), *neuron migration* (GO: 0001764), *cell adhesion* (GO: 0007155), and *synapse* (GO: 0045202).

The expression patterns of these genes was analyzed in relation to the rostrocaudally segmented domains: Pro in Ist, IPR in r1-r, and IPC in r1-c. It should be borne in mind that the IPR may be seen in both median and paramedian sections, while the Pro and IPC are best visualized in paramedian sections (schemes in Figure 1A; García-Guillén et al., 2020). In addition, we considered three deep to superficial (pial) sub-divisions within the IPR, namely the *apical* (IPRa), *intermediate* (IPRi), and *basal* (IPRb) sub-nuclei, corresponding to the RA, RDM and RCM plus RCL, respectively, in the chick IPN model (Lorente-Cánovas et al., 2012). Medio-lateral subdivisions have also been described within the IPRb and IPC but these were not considered here due to insufficient data available in the ADMBA database.

We grouped the genes with similar expression patterns in the E18.5 IPN (Supplementary Table 2) based on their distribution in the following IPN subunits or their combinations: Pro (Figures 1B,C); Pro and IPR (Figures 1D–F); Pro and IPC (Figures 1G–I); IPRa (Figures 1J–L); IPRa and IPRb (Figure 1M); IPRi (Figures 1N,O); IPRb and IPRi (Figures 2A,B); IPRb (Figure 2C); the whole IPR (Figures 2D–F); IPR and IPC (Figures 2G–I); IPC (Figures 2J–L); Pro, IPR, and IPC (Figures 2M–O).

In terms of known gene families, their members are often expressed in complementary territories of the IPN. For instance, *Sox11* and *Sox6* (Figures 1K,L) are expressed in the IPRa, *Sox14* in the IPRi and IPRb, and *Sox2* in the IPC. Similarly, *Slit1* (IPRb and IPRi) and *Slit2* (IPRa: Figure 1J) are also expressed in complementary IPR sub-divisions. On the other hand, there are also genes of the same family expressed in the same IPN territory, as is the case of *Tshz1* and *Tshz2* that are both expressed exclusively in the IPC, or *Irx2* (Figure 1F) that is expressed in the Pro along with *Irx4*, and in the entire IPR along with *Irx1* (Supplementary Table 2). Regarding other gene families relevant for neural development, such as ephrins, Ephs and cadherins, they were detected widely in the IPN since each of them had one to four members within each of the five IPN sub-divisions (Supplementary Figures 4A,B).

The IPN also displayed a regionalized distribution of some families of neurotransmitter receptors (included in GO: 0045202 in Supplementary Table 1). These mostly consisted of cholinergic (*Chrna2, Chrna4, Chrna5*, and *Chrm3*: Figure 2A and data not shown), GABAergic (*Gabra5* and *Gabrg2*) and glutamatergic receptors (*Grm4*, *Gria1, Gria3, Grik1-3*, and *Grin3a*: Figures 2F,H and data not shown). Transcripts for these three types of receptors were evident in the IPR, while the Pro expressed only cholinergic and glutamatergic receptors, and the IPC only glutamatergic receptors (Supplementary Figure 4C). In terms of neurotransmitter phenotypes, there were regionalized populations expressing genes involved in the synthesis or release of GABA, glutamate, nitric oxide or the enkephalin or nociceptin peptides (Supplementary Figure 4D).

**FIGURE 3:**
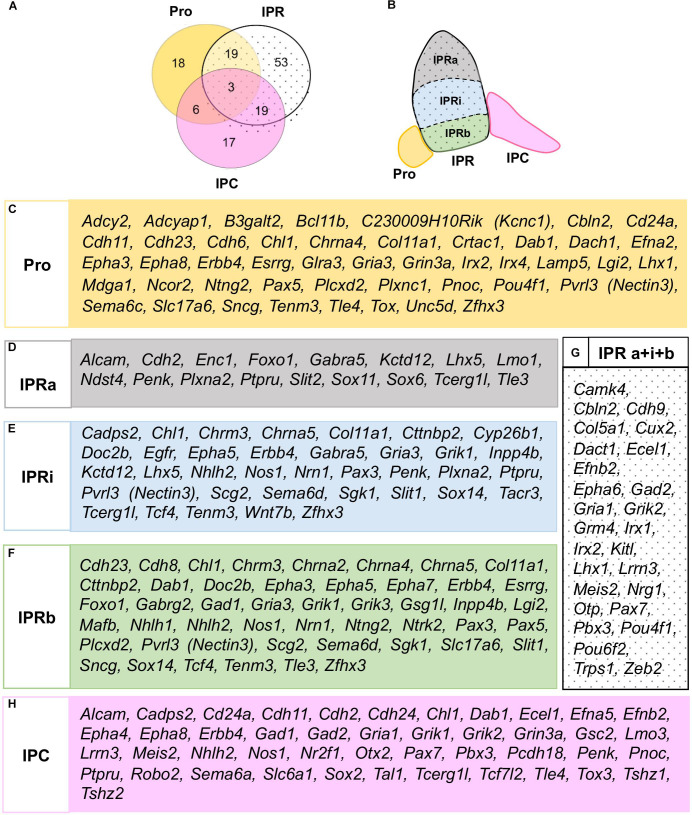
Global distribution of the regionalized genes in relation to the IPN sub-divisions. **(A)** Scheme representing the number of genes expressed in either the Pro, IPR or IPC, or combinations of these domains. **(B)** Scheme representing the rostro-caudal and deep-to-superficial sub-divisions of the IPN in colors: Pro (yellow), IPRa (gray), IPRi (blue), IPRb (green), IPC (pink). The entire IPR is filled with a dotted background. **(C–H)** Lists of the genes expressed in the Pro **(C)**, IPRa **(D)**, IPRi **(E)**, IPRb **(F)**, entire IPR **(G)**, and/or IPC **(H)**.

According to this analysis at E18.5, some genes may be specific to one of the three principal domains, while others express combinations of them (Figure 3A). There were three genes expressed in all three domains (*Chl1, Erbb4*, and *Dab1*: Figures 2M–O, 3A), although they were differentially expressed within the IPR. On the whole, and considering the further additional sub-divisions of the IPR (Figure 3B), we identified 46 genes expressed in the Pro, 17 in the IPRa, 36 in the IPRi, 47 in the IPRb and 45 in the IPC, as well as another 27 genes detected in the entire IPR (IPRa, IPRi, and IPRb: Figures 3C–H).

In general, these patterns of expression persisted at P4, with only a few genes displaying a difference in any of the sub-region between stages (Supplementary Table 3 and Supplementary Figure 3).

### Functional Categories of the Genes Expressed in the IPN

**FIGURE 4:**
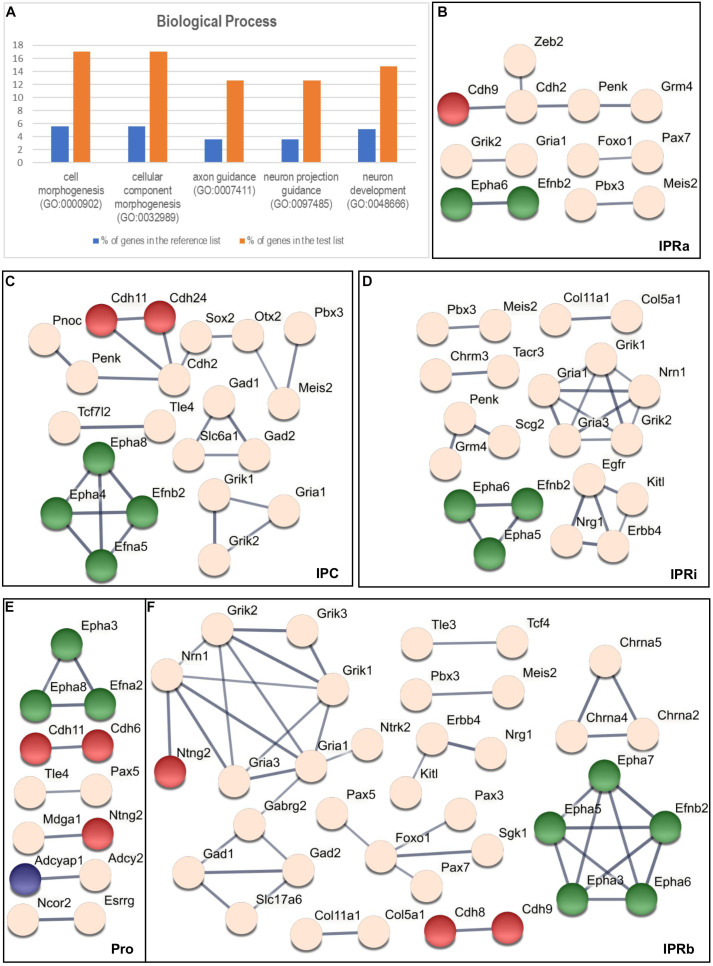
Overrepresentation test and the interaction analysis of the regionalized genes within the IPN. **(A)** Graphic results of the PANTHER Statistical Overrepresentation Test (*p* < 0.00005). For each functional category, the orange bar represents the percentage of genes in the analyzed (test) list (135 genes: see “Materials and Methods” section) that match this category, while the blue bar corresponds to the percentage of genes in the reference list (2,038 genes: see “Materials and Methods” section) associated to the same category. **(B–F)** Respective STRING networks of the genes expressed in the Pro **(E)**, IPRa **(B)**, IPRi **(D)**, IPRb **(F),** and IPC **(C)**. Each node represents a gene and each line symbolizes a predicted interaction (with a minimum required confidence score of 0.7: see “Materials and Methods” section). The thickness of the line represents the strength of the data supporting the predicted interaction. The green nodes correspond to genes that belong to every GO family represented in **(A)**, the red nodes symbolize genes included only in both the *cell morphogenesis* (GO: 0000902) and the *cellular component morphogenesis* (GO: 0032989) families, the blue nodes correspond to genes belonging exclusively to the *neuron development* family (GO: 0048666) and the light pink nodes represent genes not included in any of the GO categories listed in **(A)**.

We carried out a statistical overrepresentation test using the three PANTHER GO-slims datasets (Mi et al., 2019b) to find functional categories that might be overrepresented among the 135 genes selected relative to the starting list of 2,038 genes. Using the Biological Process dataset this analysis yielded 5 enriched GO families, which included 26 genes in total (see Figure 4A and Supplementary Table 4): *cell morphogenesis* (GO: 0000902), *cellular component morphogenesis* (GO: 0032989), *axon guidance* (GO: 0007411), *neuron projection guidance* (GO: 0097485), and *neuron development* (GO: 0048666).

The majority of these genes (17 out of 26) were common to the five GO families (*Efna2, Efna5, Efnb2, Epha3-8, Plxna2, Plxnc1, Sema6a, Sema6c, Sema6d, Slit1, Slit2, and Unc5d*), whereas another 6 genes were only included in the *cell morphogenesis* and *cellular component morphogenesis* families (*Cdh6, Cdh8, Cdh9, Cdh11, Cdh24*, and *Ntng2*) and 3 genes appeared exclusively in the *neuron development* family (*Adcyap1, Ntrk2*, and *Tenm3*).

The analysis with the Cellular Component dataset retrieved 4 overrepresented families, in which 53 of the 135 genes were included (Supplementary Table 4): *plasma membrane* (GO: 0005886), *cell periphery* (GO: 0071944), *membrane* (GO: 0016020), and *plasma membrane part* (GO: 0044459).

In turn, the overrepresentation test with the Molecular Function dataset yielded no statistically significant results (data not shown) according to the criteria defined in the “Materials and Methods” section.

### Interaction Analysis of the Genes Expressed in the IPN

We wanted to determine potential molecular interactions within each sub-division of the IPN (Figure 3B) and thus, we classified the 135 genes that mark the IPN into 5 overlapping regional groups based on their expression in the Pro (Figure 3C), IPRa (Figures 3D,G), IPRi (Figures 3E,G), IPRb (Figures 3F,G) and/or IPC (Figure 3H). This analysis generated five interactomes as outcomes (see Figures 4B–F) and to analyze these, we focused on the networks between genes belonging to the overrepresented GO families (Figures 4B–F, genes in green, red and blue). Most of the genes included were common to the five families (green), while others were only members of either the *cell morphogenesis* and *cellular component morphogenesis* families (red), or the *neuron development* family (blue). A large part of these networks involved either ephrins and Eph receptors (*Efna2, Efna5, Efnb2, Epha3, Epha4, Epha5, Epha6, Epha7, and Epha8*) or cadherin genes (*Cdh2, Cdh6, Cdh8, Cdh9, Cdh11, and Cdh24*). The other genes from the overrepresented families were *Ntng2*, which interacts with *Mdga1* in the Pro (Figure 4E) or with *Nrn1* in IPRb (Figure 4F), and *Adcyap1*, which interacts with *Adcy2* in the Pro (Figure 4E).

Additional networks involved genes not associated with the overrepresented GO families (Figures 4B–F, light pink genes), including 17 TFs and transcriptional regulators, and 12 neurotransmitter receptors (classified in GO: 0006355 and GO: 0045202, respectively, see Supplementary Table 1).

## Discussion

In this study, we retrieved 135 genes from the ADMBA database (Thompson et al., 2014) that are expressed in the E18.5 IPN in a regionalized, non-ubiquitous pattern, and that were largely conserved up to P4, supporting their involvement in IPN development at these perinatal stages.

The 135 genes were regionally expressed in the developing IPN, i.e.: they were expressed in at least one of the principal domains of the IPN (Pro, IPR, IPC). This segmentation of the IPN was initially proposed in the chick (Lorente-Cánovas et al., 2012) and later confirmed in the mouse (Moreno-Bravo et al., 2014; Ruiz-Reig et al., 2019; García-Guillén et al., 2020). Here, we further characterized the molecular profiles for each anteroposterior segment of the IPN located in their respective neuromeric units (Ist, r1-r and r1-c: Aroca and Puelles, 2005; Alonso et al., 2013). In addition, we also describe the molecular regionalization of the deep to superficial areas of the IPR (IPRa, IPRi, IPRb). To date, 5 TFs have been used as markers for diverse IPN populations: *Nkx6.1, Pax7, Otp, Otx2*, and *Irx2* (Lorente-Cánovas et al., 2012; Moreno-Bravo et al., 2014; García-Guillén et al., 2020). Remarkably, our work identified up to 50 transcriptional regulators within the IPN, as well as other genes involved in different aspects of neural development.

To obtain a more detailed functional interpretation of the results on gene expression, we performed an overrepresentation test of functional categories, as well as an analysis of putative interactions.

The statistical overrepresentation test identified families involved in different aspects of neural development, such as *axon guidance* or *cell morphogenesis*. Based on their relevance in brain development this functional overrepresentation is particularly significant since we used the preselected genes as the starting set (Thompson et al., 2014). Surprisingly, we did not obtain overrepresented families related to neuronal migration, which might have been expected considering that the IPN is formed wholly by migratory cells (Lorente-Cánovas et al., 2012). One possible explanation is that the molecules related to migration may be mainly expressed at earlier stages or alternatively, they may be expressed ubiquitously in the IPN and thus, ruled out in our screening. Indeed, the *Dcc* gene, whose signal is needed by IPN neurons to migrate, is expressed in all sub-regions of the IPN, with no evident differences between them (García-Guillén et al., 2020). Other molecules involved in migration may be downregulated once the majority of IPN neurons arrive at their final positions.

The overrepresentation of genes from the *axon guidance* and *neuron projection guidance* families (GO: 0007411, GO: 0097485) could be explained by the establishment of connections in the IPN at perinatal stages. E18.5 is a stage immediately prior to the time of axon arrival, the formation of dendritic growth cones and synaptogenesis, which begins in the first postnatal days (Lenn, 1978; Barr et al., 1987). Both of these GO families include genes expressed in cells with growing axons, in pathway cells or in the target neurons (Guan and Rao, 2003; Bellon and Mann, 2018; Russell and Bashaw, 2018). In fact, IPN genes from these overrepresented families are included in the four main families of axon guidance cues and receptors (ephrins/Ephs, Netrins/DCC-Unc5, Semaphorins/Neuropilins-Plexins, and Slits/ROBO), evidence of the importance of axon guidance mechanisms at this stage of IPN development. Each IPN subpopulation might be expected to display a differential molecular identity prior to establishing its specific incipient connections. Interestingly, projections of diverse habenular populations preferentially target specific IPN areas. For instance, the nicotinic receptor subunits *Chrna4* and *Chrna2* (Shih et al., 2014) are expressed in the MHb in different types of neurons and they project to distinct target neurons in the IPN. The *Chrna5* expressing population of the IPR in the IPN receives direct ventral MHb input and projects to other specific brainstem structures (Hsu et al., 2013). By contrast, habenular *Otx2* neurons target IPC cells that also express *Otx2* (Ruiz-Reig et al., 2019). Thus, the expression of some the genes in the IPN could be responsible for the generation of sub-circuits between neurons of the MHb, the IPN and its efferents.

The other overrepresented families of *cell morphogenesis* and *cellular component morphogenesis* (GO: 0000902, GO: 0032989) may be related to the changes in the cytoskeleton that are involved in the cell type characteristic dendritic arborization and their positioning through somata translocation. These processes could participate in the acquisition of the diverse neuronal morphologies in the different domains of this complex nucleus. In this respect, there are cytoarchitectural variations associated with different IPN sub-regions, such as diverse cell shapes (from round to elongated and bipolar neurons), as well as differences in cell size and packing (Hemmendinger and Moore, 1984). Regarding the Cellular Component dataset, the families overrepresented are related to the cell surface and plasma membrane, such that they might reflect a morphological correlate for the axon guidance and cell morphogenesis functional families mentioned above.

The interactomes predicted by our analysis included several networks that mainly involved the ephrin, Eph, and cadherin genes, as well as several TFs and neurotransmitter receptors. These networks highlighted the principal intra-familiar interactions of ephrins, Ephs, and cadherins. The lack of further inter-familiar connections may be due to the high stringency conditions used to generate the interactome. On the other hand, these networks include intercellular (e.g., between ephrin and Eph receptors) as well as intracellular (e.g., involving TFs) interactions. In this respect, a limitation of our study was that the material did not allow to discern the eventual co-expression of these interacting genes at the cellular level.

Ephrins and Ephs have been associated with numerous activities, such as axon guidance, synaptic plasticity, topographical ordering in synaptogenesis and cell migration (Kullander and Klein, 2002). Indeed, they are involved in the formation of the anterior commissure, the corticospinal tract and the corpus callosum (Kullander and Klein, 2002; Reber et al., 2007). The retroflex afferents of the IPN typically (and uniquely) cross the midline of the complex repeatedly, making multiple *en passant* synapses (Cajal, 1911). This process may involve ephrin/Eph signaling given their roles in axon guidance and synapse development. These molecules also have a preponderant role in the establishment of topographic maps, such as the retinotopic mapping (McLaughlin and O’Leary, 2005) or the formation of thalamocortical connections (Gezelius and López-Bendito, 2017). Therefore, they could also be involved in the generation of topographic maps in the IPN. In relation to this model, Ephrins and Ephs could also influence neuronal migration taking into account that they regulate such processes in the cortex and olfactory bulb (Rodger et al., 2012).

Cadherins also participate in the formation of neuronal connections, since combinatorial expression of cadherins in the growth cone favor specific interactions with neuronal targets expressing the same cadherin set, participating in the establishment of retinotectal projections and other axon guidance events (Ranscht, 2000; Redies and Puelles, 2001). Therefore, these adhesion molecules could be involved in the generation of specific neuronal sub-circuits between the MHb and the IPN, as described for cholinergic receptors and Otx2 above. Interestingly, *Cdh6*, *Cdh8* and *Cdh11* are also expressed in the MHb at E18.5 and P4, as evident in the ADMBA database (data not shown: see also Korematsu and Redies, 1997), and they could participate in forming local connections between IPN sub-regions (Quina et al., 2017). Significantly, cadherins are also involved in neuronal migration, for example in the tangential migration of facial branchiomotor neurons (Ju et al., 2004) and precerebellar neurons in the caudal hindbrain (Taniguchi et al., 2006).

Thus, both ephrin/Ephs and cadherins participate in processes of axon guidance and cell migration, together with other known gene families like Netrin-1/DCC, Slit/ROBO and immunoglobulins (Brose and Tessier-Lavigne, 2000; Maness and Schachner, 2007). In relation to the MHb-IPN system, Netrin-1/DCC is involved in both IPN neuronal migration (García-Guillén et al., 2020) and axon guidance in the fasciculus retroflexus (Belle et al., 2014). From the predicted interactomes, the signaling mediated by Nrg1/Erbb4 is noteworthy since it participates in the migration of neuroblasts toward the olfactory bulb (Anton et al., 2004), as well as in the migration of neurons from the basal ganglia to the cortex, simultaneously guiding thalamocortical projections (López-Bendito et al., 2006). Another predicted interaction of interest from our model is that of *Ntng2* and *Nrn1*, since both are also involved in neuronal migration and axon guidance (Zhou and Zhou, 2014; Heimer et al., 2020). Therefore, these signaling systems could eventually participate in the migration and arrangement of different IPN populations, and in the subsequent establishment of their connections.

The interactomes included TFs that would be expected to regulate the expression of genes involved in axon guidance and neuronal migration (Russell and Bashaw, 2018). Indeed, two TFs (*Sox2* and *Zeb2*) shared their networks with *Cdh2*. Other interactions between TFs and the genes mentioned above may be expected, although they may not have appeared due to the high stringency of our analysis. Other members of the interactomes were genes related to neurotransmitters that are known to regulate axon guidance in some systems (Ruediger and Bolz, 2007).

The neurotransmitter phenotype described in the adult IPN mainly comprises of GABAergic cells in all sub-nuclei, with glutamatergic cells mostly limited to the IPR and partially in the IPC (Quina et al., 2017). This pattern is largely visible at P4 and in addition, our data point to the presence of glutamatergic neurons in the Pro. In terms of the enkephalin producing cells identified by *Penk*, our results show that this gene is expressed in the IPR and partially in the IPC, with some changes in the IPR from E18.5 to P4 where its distribution is similar to that observed at early postnatal stages in the rat (Morita et al., 1990). *Pnoc* is a marker for nociceptin producing neurons and its expression also changes in the IPN from E18.5 to P4. Hence, it appears that the acquisition of neurotransmitter phenotypes is dynamic at these developing stages, at least regarding these neuropeptides.

In conclusion, the results from both the predicted interactomes and the categories of overrepresented functions together indicate that genes active in processes like axon guidance or neuronal development and morphogenesis, would be relevant to the formation and connectivity of the IPN. In addition, the molecular identities of the IPN sub-regions have been further characterized here, adding new knowledge to their previous histological and chemoarchitectonic definitions. Hence, the IPN model proposed integrates data on gene expression and functionality, establishing a possible base for further studies into the formation and function of the IPN, and eventually, into the neurological disorders associated with this structure.

## Data Availability Statement

Publicly available datasets were analyzed in this study. This data can be found here: https://developingmouse.brain-map.org/.

## Ethics Statement

The animal study was reviewed and approved by the University of Murcia Ethical Committee for Animal Experimentation. Concerning the material shown in Supplementary Figure 1, mice were maintained according to European Union guidelines (2010/63/EU), Spanish law (Royal Decree 53/2013 and the Royal Decree 1386/2018) regarding the care and handling of research animals.

## Author Contributions

PA and FM conceived and designed the research. IG-G performed the data mining. IG-G, FM, and PA analyzed the data and wrote the manuscript. IG-G and AA performed the image analysis and figure preparation. LP gave general advice and edited the manuscript. All authors contributed to the article and approved the final submitted version.

## Conflict of Interest

The authors declare that the research was conducted in the absence of any commercial or financial relationships that could be construed as a potential conflict of interest.

## Footnotes

1 https://developingmouse.brain-map.org/

2 https://ensembl.org/

3 http://geneontology.org/

4 http://pantherdb.org/

5 https://string-db.org/
