# Supplementary material for: Multiple Regionalized Genes and Their Putative Networks in the Interpeduncular Nucleus Suggest Complex Mechanisms of Neuron Development and Axon Guidance
Source: Front Neuroanat. 2021 Feb 16;15:643320. doi: 10.3389/fnana.2021.643320 (PMC7921722; doi:10.3389/fnana.2021.643320)
Supplement: Supplementary file 1 [file Image_1.pdf]

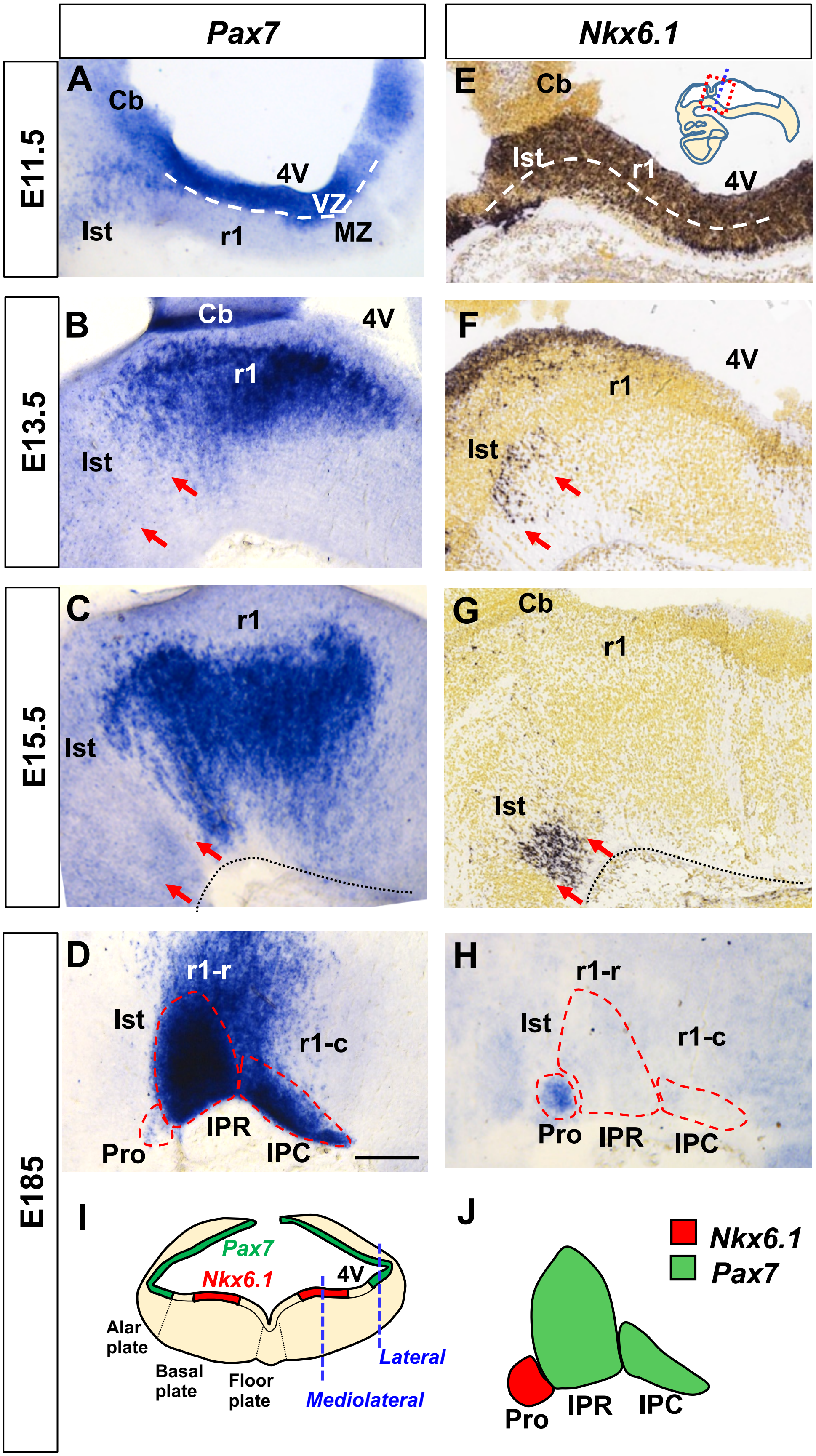

Supplementary Figure 1**. Origin and final fate of *Pax7*⁺ and *Nkx6.1*⁺ IPN populations.** In situ hybridization of *Pax7* (A-D) and *Nkx6.1* (E-H) in parasagittal sections (from region boxed in E) at E11.5 (A, E), E13.5 (B, F), E15.5 (C, G) and E18.5 (D, H). (I) Schematic cross-section showing the origin of *Pax7* in the alar VZ and *Nkx6.1* in the basal VZ. (J) Sagittal diagram of the IPN indicating the distribution of *Pax7* (green territory) and *Nkx6.1* (red territory) at E18.5. Red arrows delimitate the isthmic territory. Cb: cerebellum; VZ: ventricular zone; MZ: mantle zone; 4V: fourth ventricle. The rest of abbreviations used are as specified in the main text. The ISH for A-D and H was performed according to the protocol described in García-Guillén et., 2020. Scale bars=200μm.
