# Supplementary material for: Multiple Regionalized Genes and Their Putative Networks in the Interpeduncular Nucleus Suggest Complex Mechanisms of Neuron Development and Axon Guidance
Source: Front Neuroanat. 2021 Feb 16;15:643320. doi: 10.3389/fnana.2021.643320 (PMC7921722; doi:10.3389/fnana.2021.643320)
Supplement: Supplementary file 2 [file Image_2.pdf]

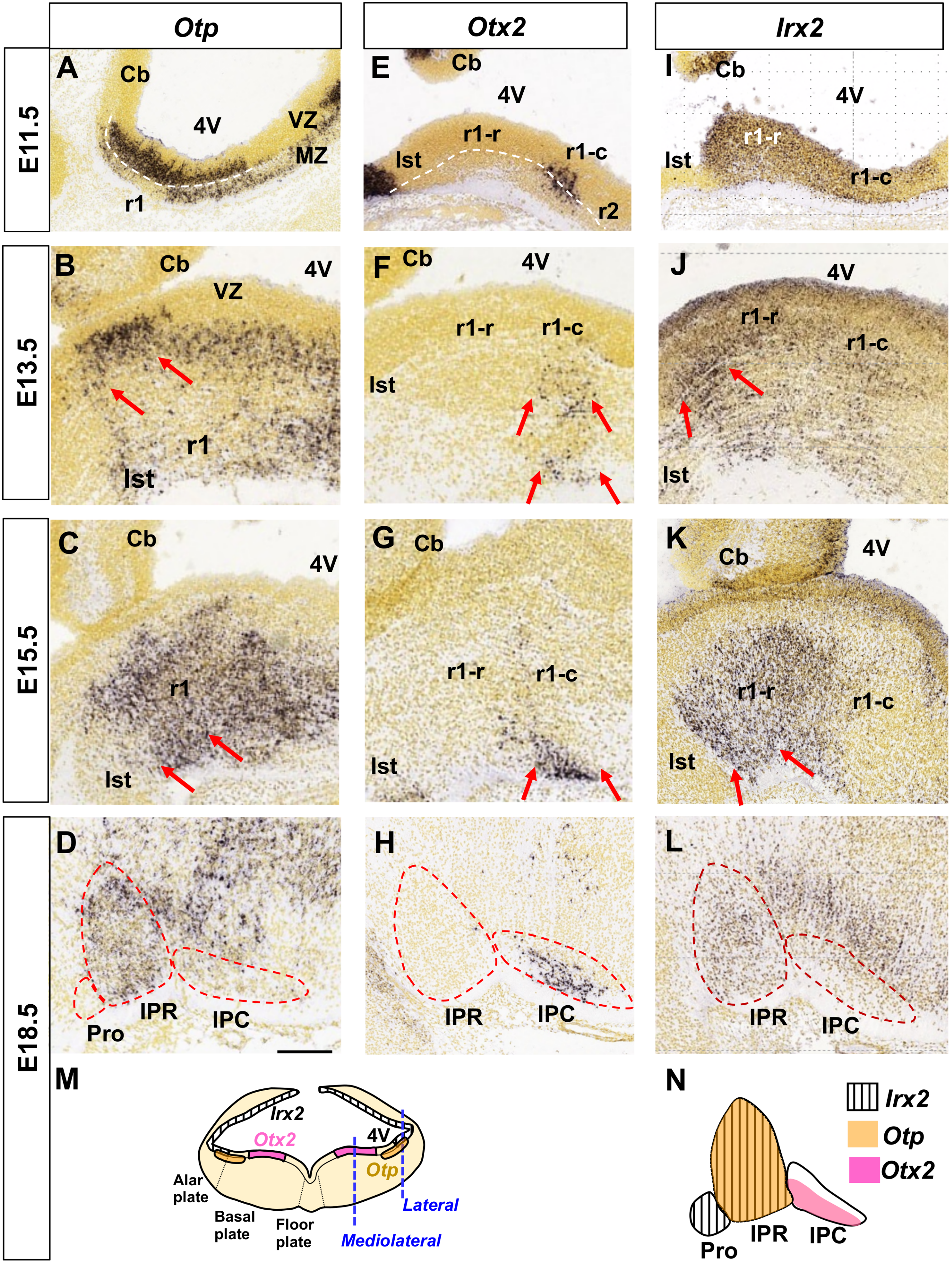

***Supplementary Figure 2*. Origin and final fate of *Otp*⁺, *Otx2*⁺ and *Irx2*⁺ IPN populations.** In situ hybridization of *Otp* (A-D), *Otx2* (E-H) and *Irx2* (I-L) in parasagittal sections at E11.5 (A, E, I), E13.5 (B, F, J), E15.5 (C, G, K) and E18.5 (D, H, L). Red arrows point to the migratory populations. (M) Schematic cross-section showing the origin of *Irx2*, *Otx2* and *Otp* in the alar, basal and alar/basal VZ, respectively. (N) Sagittal diagram of the IPN indicating the distribution of *Irx2* (striped territory), *Otx2* (pink territory) and *Otp* (yellow territory) at E18.5. Cb: cerebellum; VZ: ventricular zone; MZ: mantle zone; 4V: fourth ventricle. The rest of abbreviations used are as specified in the main text. Scale bars=200µm.
