# Supplementary material for: Multiple Regionalized Genes and Their Putative Networks in the Interpeduncular Nucleus Suggest Complex Mechanisms of Neuron Development and Axon Guidance
Source: Front Neuroanat. 2021 Feb 16;15:643320. doi: 10.3389/fnana.2021.643320 (PMC7921722; doi:10.3389/fnana.2021.643320)
Supplement: Supplementary file 3 [file Image_3.pdf]

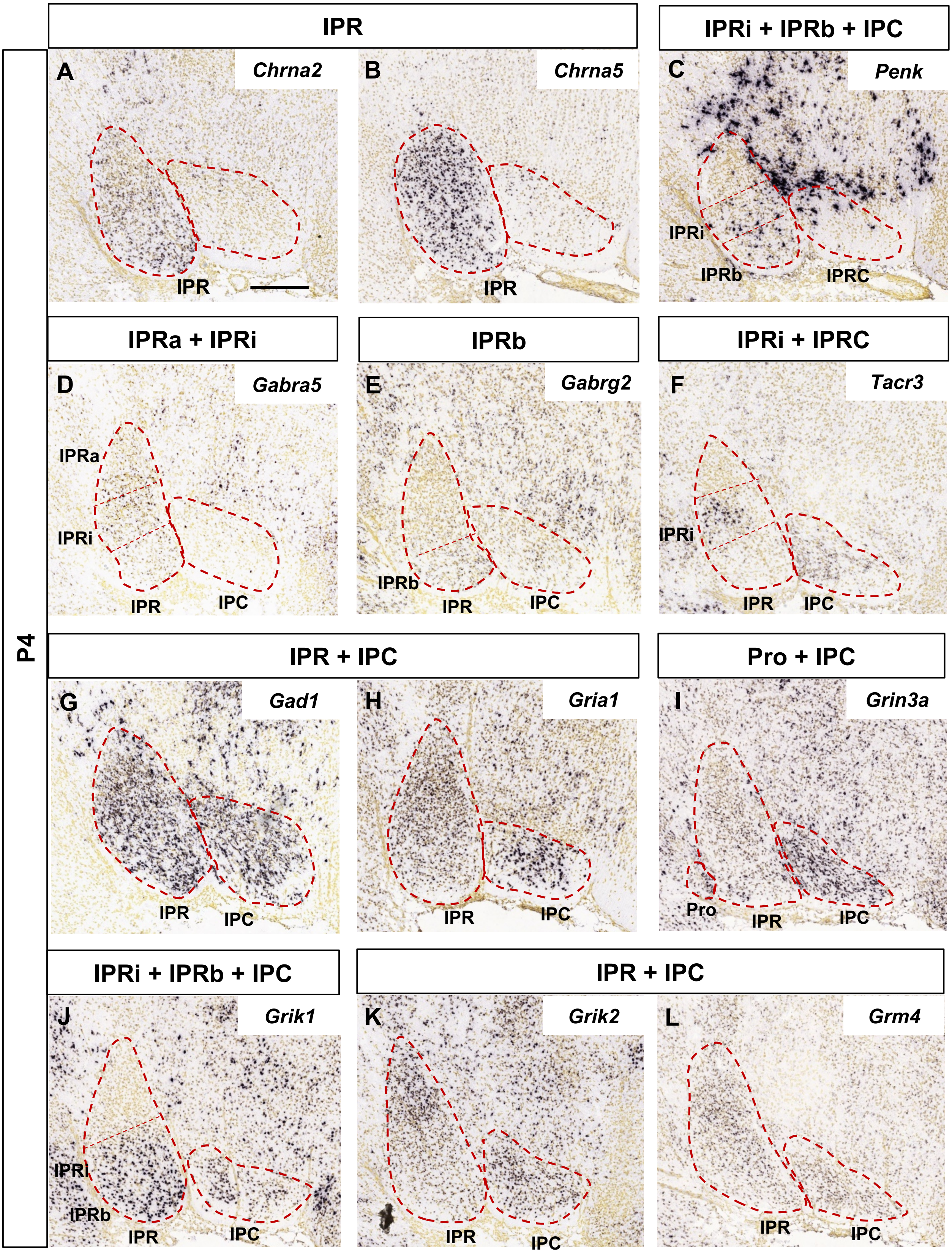

Supplementary Figure 3. **Expression of regionalized genes in the IPN at P4.** (A-L) Representative parasagittal sections displaying the expression of genes related to neurotransmission. The different expression patterns, corresponding to respective combinations of IPN subdivisions, are defined above the images. The abbreviations used are as specified in the main text. Scale bars= 200 μm.
