# Supplementary material for: Multiple Regionalized Genes and Their Putative Networks in the Interpeduncular Nucleus Suggest Complex Mechanisms of Neuron Development and Axon Guidance
Source: Front Neuroanat. 2021 Feb 16;15:643320. doi: 10.3389/fnana.2021.643320 (PMC7921722; doi:10.3389/fnana.2021.643320)
Supplement: Supplementary file 4 [file Image_4.pdf]

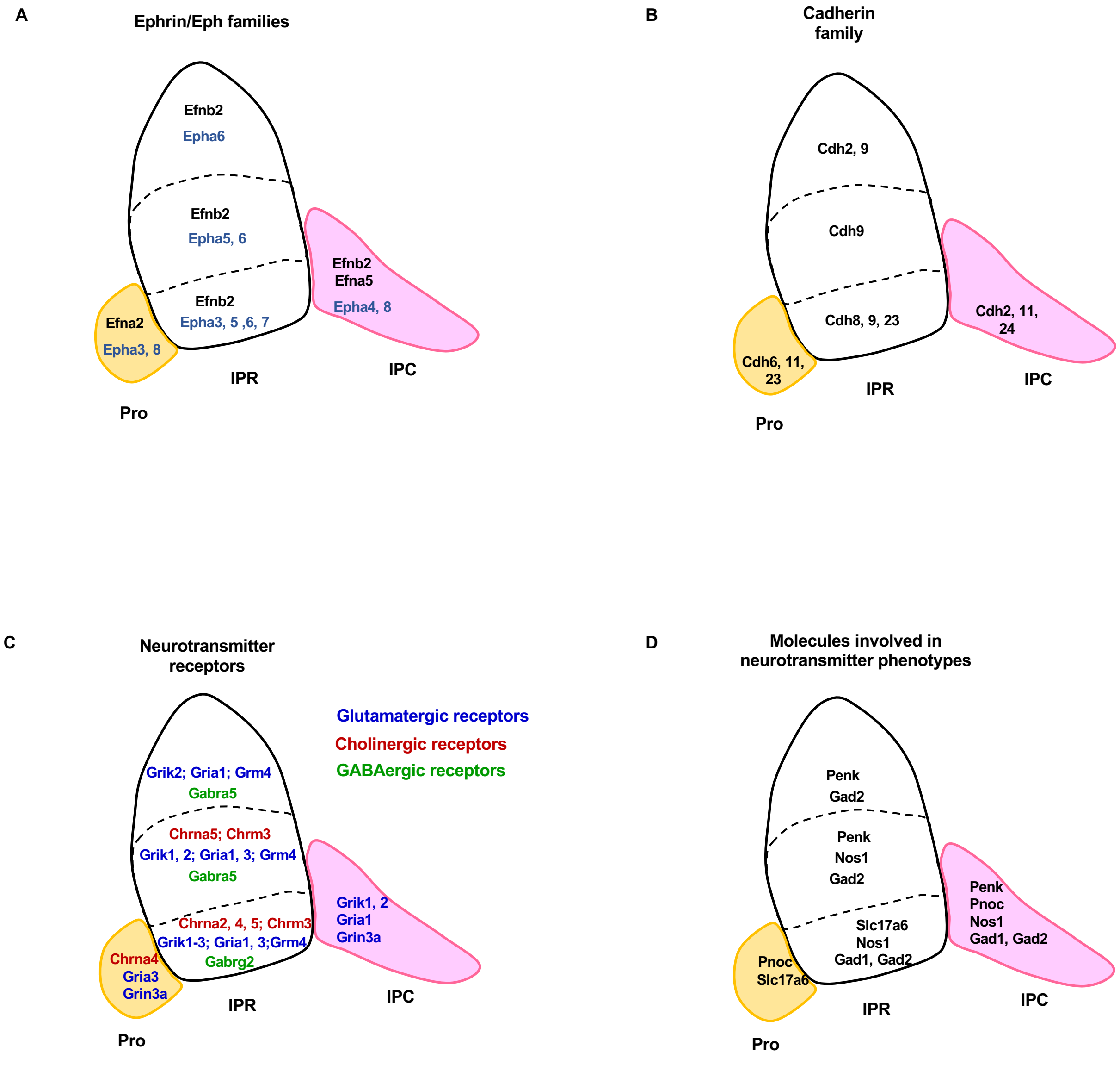

Supplementary Figure 4. Sagittal diagrams showing the expression of regionalized genes of ephrin/Eph (A) and cadherin (B) families, as well as those encoding neurotransmitter receptors (C) and molecules involved in neurotransmitter phenotypes (D), at E18.5. In D, Gad1 and Gad2 enzymes are markers for GABAergic cells, Slc17a6 is a glutamate presynaptic transporter in glutamatergic cells, *Nos1* encodes nitric oxide synthase 1, and *Penk* and *Pnoc* encode the precursors of enkephalin and nociceptin neuropeptides, respectively.
