# Supplementary material for: Multiple Regionalized Genes and Their Putative Networks in the Interpeduncular Nucleus Suggest Complex Mechanisms of Neuron Development and Axon Guidance
Source: Front Neuroanat. 2021 Feb 16;15:643320. doi: 10.3389/fnana.2021.643320 (PMC7921722; doi:10.3389/fnana.2021.643320)
Supplement: Supplementary file 5 [file Data_Sheet_1.PDF]

| Gene Ontology (GO) term and accession number | Genes |
|---|---|
| **Regulation of transcription, DNA-templated (GO: 0006355)** | *Adcyap1, Bcl11b, Camk4, Cux2, Dach1, Dact1, Egfr, Epha5, Erbb4, Esrrg, Foxo1, Gsc2, Irx1, Irx2, Irx4, Lhx1, Lhx5, Lmo1, Mafb, Meis2, Ncor2, Nhlh1, Nhlh2, Nos1, Nr2f1, Nrg1, Otp, Otx2, Pax3, Pax5, Pax7, Pbx3, Pou4f1, Pou6f2, Sox11, Sox14, Sox2, Sox6, Tal1, Tcf4, Tcf7l2, Tle3, Tle4, Tox, Tox3, Trps1, Tshz1, Tshz2, Zeb2, Zfhx3* |
| **Neuron projection guidance (GO: 0097485)** | *Alcam, Bcl11b, Chl1, Efna2, Efna5, Efnb2, Epha4, Epha5, Epha6, Epha7, Epha8, Lhx1, Otx2, Plxna2, Plxnc1, Robo2, Sema6a, Sema6c, Sema6d, Slit1, Slit2, Unc5d, Wnt7b* |
| **Neuron migration (GO: 0001764)** | *Chl1, Dab1, Erbb4, Lhx1, Mdga1, Nrg1, Ntng2, Ntrk2, Sema6a, Sox14, Unc5d* |
| **Cell adhesion (GO: 0007155)** | *Alcam, Cd24a , Cdh11, Cdh2, Cdh23, Cdh24, Cdh6, Cdh8, Cdh9, Chl1, Col5a1, Dab1, Efna5, Efnb2, Egfr, Epha3, Epha4, Epha5, Epha7, Epha8, Kitl, Mdga1, Ntng2, Pcdh18, Plxna2, Plxnc1,Pvrl3 (Nectin3), Ptpru, Sema6a, Slit2, Sox2, Tenm3, Unc5d, Wnt7b* |
| **Synapse (GO: 0045202)** | *Adcyap1, Cadps2, Camk4, Cbln2, Cd24a, Cdh11, Cdh2, Cdh23, Cdh8, Cdh9, Chrm3, Chrna2, Chrna4, Chrna5, Cttnbp2, Dab1, Dact1, Doc2b, Efna2, Efna5, Efnb2, Egfr, Epha4, Epha7, Erbb4, Gabra5, Gabrg2, Gad1, Gad2, Glra3, Gria1, Gria3, Grik1, Grik2, Grik3, Grin3a, Gsg1l, C230009H10Rik (Kcnc1), Lamp5, Mdga1, Pvrl3, Nos1, Nrg1, Nrn1, Ntng2, Ntrk2, Penk, Pnoc, Slc17a6, Slc6a1, Sncg* |
| **Other** | *Adcy2, B3galt2, Col11a1, Crtac1, Cyp26b1, Ecel1, Enc1, Grm4, Inpp4b, Kctd12, Lgi2, Lmo3, Lrrn3, Ndst4, Plcxd2, Scg2, Sgk1, Tacr3, Tcerg1l* |

***Supplementary Table 1.*** GO classification of the genes regionalized within the IPN.

| | |
|---|---|
| **Pro** | *Adcy2, Adcyap1, B3galt2, Bcl11b, C230009H10Rik (Kcnc1), Cdh6, Crtac1, Dach1, Efna2, Glra3, Irx4, Lamp5, Mdga1, Ncor2, Plxnc1, Sema6c, Tox, Unc5d* |
| **IPRa** | *Enc1, Lmo1, Ndst4, Slit2, Sox11, Sox6* |
| **IPRi** | *Cyp26b1, Egfr, Tacr3, Wnt7b* |
| **IPRb** | *Cdh8, Chrna2, Epha7, Gabrg2, Grik3, Gsg1l, Mafb, Nhlh1, Ntrk2* |
| **IPRa + IPRi** | *Gabra5, Kctd12, Lhx5, Plxna2* |
| **IPRb + IPRi** | *Chrm3, Chrna5, Cttnbp2, Doc2b, Epha5, Inpp4b, Nrn1, Pax3, Scg2, Sema6d, Sgk1, Slit1, Sox14, Tcf4* |
| **IPRa + IPRb** | *Foxo1, Tle3* |
| **IPR** | *Camk4, Cdh9, Col5a1, Cux2, Dact1, Epha6, Grm4, Irx1, Kitl, Nrg1, Otp, Pou6f2, Trps1, Zeb2* |
| **IPC** | *Cdh24, Efna5, Epha4, Gsc2, Lmo3, Nr2f1, Otx2, Pcdh18, Robo2, Sema6a, Slc6a1, Sox2, Tal1, Tcf7l2, Tox3, Tshz1, Tshz2* |
| **Pro + IPRi + IPRb** | *Col11a1, Gria3, Pvrl3 (Nectin3), Tenm3, Zfhx3* |
| **Pro + IPRb** | *Cdh23, Chrna4, Epha3, Esrrg, Lgi2, Ntng2, Pax5, Plcxd2, Slc17a6, Sncg* |
| **Pro + IPR** | *Cbln2, Irx2, Lhx1, Pou4f1* |
| **Pro + IPC** | *Cd24a, Cdh11, Epha8, Grin3a, Pnoc, Tle4* |
| **IPRa + IPC** | *Alcam, Cdh2* |
| **IPRa + IPRi + IPC** | *Penk, Ptpru, Tcerg1l* |
| **IPRi + IPC** | *Cadps2* |
| **IPRb + IPRi + IPC** | *Grik1, Nhlh2, Nos1* |
| **IPRb + IPC** | *Gad1* |
| **IPR + IPC** | *Ecel1, Efnb2, Gad2, Gria1, Grik2, Lrrn3, Meis2, Pax7, Pbx3* |
| **Pro+ IPRi + IPRb + IPC** | *Chl1, Erbb4* |
| **Pro+ IPRb + IPC** | *Dab1* |

***Supplementary Table 2.*** Summary of the regionalized expression of the 135 genes in the IPN at E18.5. Pro: prodromal nucleus; IPR: rostral interpeduncular nucleus; IPC: caudal interpeduncular nucleus; IPRa: apical subnucleus of IPR; IPRi: intermediate subnucleus of IPR; IPRb: basal subnucleus of IPR.

| PANTHER datasets | GO families | Genes |
|---|---|---|
| **Biological Process** | ***cell morphogenesis* (GO:0000902)** ***cellular component morphogenesis* (GO:0032989)** ***axon guidance* (GO:0007411)** ***neuron projection guidance* (GO:0097485)** ***neuron development* (GO:0048666)** | *Efna2, Efna5, Efnb2, Epha3, Epha4, Epha5, Epha6, Epha7, Epha8, Plxna2, Plxnc1, Sema6a, Sema6c, Sema6d, Slit1, Slit2, Unc5d* |
| | ***cell morphogenesis* (GO:0000902)** ***cellular component morphogenesis* (GO:0032989)** | *Cdh6, Cdh8, Cdh9, Cdh11, Cdh24, Ntng2* |
| | ***neuron development* (GO:0048666)** | *Adcyap1, Ntrk2, Tenm3* |
| **Cellular Component** | ***plasma membrane* (GO:0005886)** ***cell periphery* (GO:0071944)** ***membrane* (GO:0016020)** ***plasma membrane part* (GO:0044459)** | *Adcy2, Adcyap1, Cdh11, Cdh24, Cdh6, Cdh8, Cdh9, Chrm3, Chrna2, Chrna4, Chrna5, Egfr, Epha3, Epha4, Epha5, Epha6, Epha7, Epha8, Erbb4, Gabra5, Gabrg2, Glra3, Gria1, Gria3, Grik1, Grik2, Grik3, Grm4, Kcnc1, Ntrk2, Pcdh18, Penk, Plxna2, Plxnc1, Pnoc, Sema6a, Sema6c, Sema6d, Slc6a1, Tacr3, Tenm3* |
| | ***plasma membrane* (GO:0005886)** ***cell periphery* (GO:0071944)** ***membrane* (GO:0016020)** | *B3galt2, Cux2, Efna2, Efna5, Efnb2, Grin3a, Gsg1l, Kitl, Lamp5, Ndst4, Nos1, Slc17a6* |

***Supplementary Table 4*. Results from the overrepresentation test with PANTHER**. With the Biological Process dataset, the majority of genes (17 out of 26) were common to the five overrepresented GO families (*cell morphogenesis, cellular component morphogenesis, axon guidance, neuron projection guidance* and *neuron development*). On the other hand, other 6 genes were included only in the *cell morphogenesis* and *cellular component morphogenesis* families and 3 genes were exclusive in *neuron development*. With the Cellular Component dataset, most of the genes (41 out of 53) were common to the four overrepresented GO families (*plasma membrane, cell periphery, membrane, plasma membrane part*) whereas 12 genes belonged to *plasma membrane, cell periphery* and *membrane* families.
